# Cotranslational Folding and “Constrained Monomers” in the Maturation of HIV-1 Protease

**DOI:** 10.1016/j.jmb.2026.169788

**Published:** 2026-04-01

**Authors:** Justin M. Westerfield, Felix Nicolaus, Ronald Swanstrom, Gunnar von Heijne

**Affiliations:** 1 - **Department of Biochemistry and Biophysics**, Stockholm University, Stockholm, Sweden; 2 - **Department of Biochemistry and Biophysics**, University of North Carolina at Chapel Hill, Chapel Hill, NC, United States of America; 3 - **Lineberger Comprehensive Cancer Center**, University of North Carolina at Chapel Hill, Chapel Hill, NC, United States of America; 4 - **Science for Life Laboratory**, Stockholm University, Solna, Sweden

**Keywords:** HIV-1 protease, cotranslational proteolysis, force profile analysis

## Abstract

HIV-1 particle formation and release occur with oligomerization of Gag polyprotein precursor and budding through the cellular plasma membrane. Maturation to an infectious virion depends on multiple proteolytic cleavages of the viral polyproteins by the viral protease, PR. PR is part of the Gag-Pro-Pol polyprotein, a minor frameshifted translational variant of the Gag protein that is incorporated in the budding virion with Gag. PR is active as a dimer and must exist both in an active form in the context of the Gag-Pro-Pol precursor and as the mature dimer. Here we study the cotranslational folding of the PR monomer within frameshifted transframe-protease-reverse transcriptase (TF-PR-RT) constructs by *in vitro* translation to explore early steps of PR folding and activation. We demonstrate cotranslational folding of ribosome-bound PR at its conserved α-helix near the C-terminus. The experimental design included constructs that were either released from the ribosome, or retained on the ribosome by a translational arrest peptide constraining the PR domain to a monomeric state. Unexpectedly, we find that released TF-PR-RT dimers are refractory to cleavage by PR, while ribosome-bound monomeric chains are efficiently cleaved. We suggest that the “constrained isolation” of PR monomers on the ribosome in this system is analogous to PR monomers entering the budding virion in the context of the Gag-Pro-Pol precursor. These observations suggest a model for virion maturation in which dimerization of a subset of Pro-Pol precursors initiates cleavage of PR monomers that then dimerize and carry out most of the proteolytic processing needed for virion maturation.

## Introduction

Most retroviruses, including human immunodeficiency virus type 1 (HIV-1), assemble their virions at the plasma membrane of the infected cell [1]. The precursor proteins Gag and Gag-Pro-Pol oligomerize on the inner face of the plasma membrane to nucleate budding of the enveloped particle [2]. The Gag-Pro-Pol precursor is formed via a −1 frameshifting event that replaces the C-terminal domain of Gag with the transframe (TF) region in the −1 reading frame which allows translation to continue to include the Pro and Pol coding domains. The frameshift, however, is infrequent in that the Gag precursor is present in a 20-fold excess over the Gag-Pro-Pol precursor [3,4].

Virus particle production occurs with the Gag and Gag-Pro-Pol precursors (or even with Gag alone) but such particles are not infectious until further maturation occurs. Both precursor proteins must undergo proteolytic processing by the encoded viral protease (PR, representing the Pro portion of Gag-Pro-Pol) to produce the mature proteins required for the formation of an infectious virion, including the mature forms of the viral enzymes (reverse transcriptase (RT) and integrase (IN)) that function in the next infected cell [5]. Processing appears to be initiated in the context of virus budding and completed in the budded particle [6–8].

The Gag precursor encodes the structural proteins of the virus particle as well as the C-terminal p6 “late domain” that interacts with the cellular ESCRT proteins to effect budding and scission of the host membrane to release the viral particle [9,10]. The Gag-Pro-Pol precursor originates from the same genetic material, but does not include the late domain due to the frameshifting event that occurs upstream of the p6 coding region. The Gag-Pro-Pol precursor then enters the budding virion via interactions with Gag. Proteolytic processing of both precursors is an essential step in the virus life cycle, so much so that antiviral protease inhibitors can effectively inhibit HIV-1 maturation [11,12]. PR, which is responsible for these processing events, is an aspartic proteinase and obligate homodimer [5,13]. Thus, initial activation of protease activity requires dimerization of the Gag-Pro-Pol precursor, a process that may be enhanced by dimerization of the RT domain in the precursor [14].

There is evidence that Gag precursors can form low-order oligomers in the cytoplasm before moving to the plasma membrane where they oligomerize to induce budding [15–23]. If Gag-Pro-Pol also participates in these low-order oligomers through their Gag domains, this would limit the chance of forming Gag-Pro-Pol homodimers in favor of Gag/Gag-Pro-Pol heterodimers, thus precluding PR dimerization and activation prior to membrane-associated oligomerization and budding. The rescue of a membrane-targeting mutant of Gag-Pro-Pol by wild-type Gag is consistent with an interaction prior to reaching the membrane [24,25]. The absence of protease activation from dimerization in the cytoplasm can be inferred by the observation that if a head-to-tail PR dimer is encoded in the Gag-Pro-Pol precursor, it efficiently initiates premature proteolytic processing [26]. In addition, when a tight-binding, non-nucleoside reverse transcriptase inhibitor (NNRTI) is used to potentiate dimerization of the RT domain (encoded in the Pol portion of Gag-Pro-Pol) this also prematurely activates the viral protease [27–31]. This NNRTI-induced premature dimerization appears to happen only after the Gag-Pro-Pol precursor has migrated to the membrane where Gag and Gag-Pro-Pol engage in higher order oligomerization [32]. When premature activation does occur, it leads to a nonproductive pathway for virion formation.

The protease becomes active in the context of a dimerized Gag-Pro-Pol precursor. The Pro-Pol domain is released from the upstream portion of Gag by a cleavage at the same site that is first cleaved in Gag (SP1/NC) [33,34]. This cleavage event is followed by cleavage of a site within the TF region upstream of the PR domain [33,35–39]; importantly, evidence from an *in vitro* translation system indicates that these two upstream cleavage events occur in *cis* by the embedded dimerized protease [34].

Recognition that PR can exist as a monomer (initially in the Gag-Pro-Pol precursor) and as a dimer has led to studies of the monomeric PR structure [40–42]. Also, while the Pro-Pol domains can dimerize in their precursor form, how this structure proceeds to give mature PR (the dimer of identical 99 amino acid subunits that can then carry out cleavage at five sites in Gag and four sites in Pro-Pol) is an active area of research. The TF region, the −1 reading frame of Gag present upstream of PR, has been suggested to inhibit protease activity until it is cleaved from the N-terminus of PR [38,43,44]. One line of evidence has described flexibility within the PR structure that allows the N-terminal protease cleavage site to be cleaved in *cis* followed by cleavage of the C-terminal protease cleavage site as the mechanism of generation of mature protease from the precursor [44–48]. N-terminal extensions destabilize the PR dimer [44,45], suggesting that it is the mature PR that is responsible for most of the catalytic activity in the virion. Despite these lines of evidence, dimerization-induced activation of PR within the Gag-Pro-Pol precursor and the potential cotranslational folding of PR itself is a process that is incompletely understood.

In this report we have used Force Profile Analysis (FPA) in an *in vitro* translation system to examine cotranslational protein folding of PR, measured as the ability of the folding reaction to exert a pulling force on a downstream translational arrest peptide [49]. We find that the PR domain embedded in a truncated TF-PR-RT context has little structure until the C-terminal α-helix of PR emerges from the ribosome exit tunnel. The formation of the α-helix does not appear to nucleate upstream folding, as the folding force was unchanged when mutations to the hydrophobic core of PR were made. Longer constructs requiring a long time before release from the ribosome or that include the translation arrest signal create ribosome-attached, constrained monomers, analogous to PR in the Gag-Pro-Pol precursor. Unexpectedly, we find that only ribosome-bound, presumably monomeric, TF-PR-RT chains can be cleaved by PR, while released, presumably dimerized, chains are largely refractory to proteolysis. Viewing these findings in the context of the infected cell, we propose a model for PR activation during virion formation where infrequent precursor dimerization events during budding generate TF-Pro-Pol dimers that are cleaved from Gag and thus released from the viral envelope membrane; the released dimerized precursors then cleave proximal PR monomers in other Gag-Pro-Pol precursors in *trans* allowing the released PR monomers to then dimerize to become the highly active form of PR for virion maturation.

## Results

### Force profile analysis

FPA takes advantage of the fact that translational arrest peptides (APs) can induce a temporary pause in translation by binding tightly deep in the ribosome exit tunnel, close to the peptidyl transferase center (PTC) [50,51]. AP-induced ribosome stalling can be overcome by pulling forces acting on the nascent chain [52,53], with different APs being sensitive to different force levels [54]. Such pulling forces can be generated by, *e.g.*, cotranslational protein folding or cotranslational insertion of transmembrane segments into a membrane [49,55,56]. Therefore, APs can be conveniently used as force sensors to study cotranslational events *in vitro* and *in vivo*.

**Figure 1.**
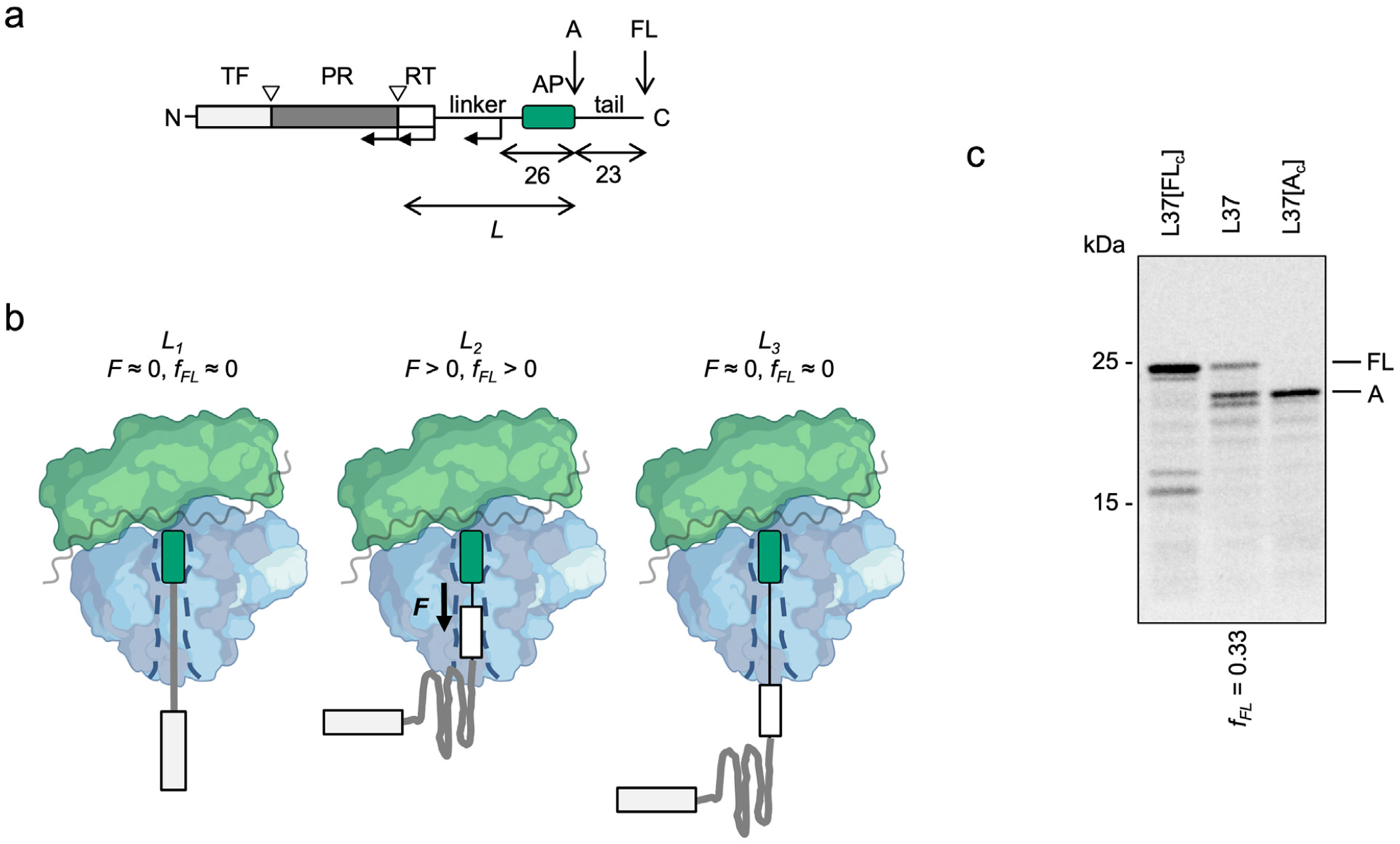
The force profile assay. (a) Basic construct (*L* = 60 residues). The HIV-1 transframe (TF; 57 residues), protease (PR; 99 residues), reverse transcriptase residues 1–30 (RT), linker, SecM(*Ec*) AP, and C-terminal tail are indicated, together with the arrested (*A*) and full-length (*FL*) products. The TF/PR and PR/RT cleavage sites are indicated by triangles. *L* denotes the number of residues between the C-terminal end of PR and the last residue in the AP. The 23-residue tail and a 26-residue segment including the AP have the same sequence in all constructs. Shorter constructs were made by progressively deleting residues in, first, the RT, then the linker, and last the C-terminal PR regions (angled arrows), as detailed in Supplementary Figure S1. (b) At construct length *L*_1_, PR (gray) is located too deep in the exit tunnel to be able to fold, no pulling force *F* is generated, and little full-length product is produced (*F* ≈ 0, *f*_*FL*_ 0). At *L*_2_, PR is starting to fold, generating a strong pulling force and more full-length product (*F* > 0, *f_FL_* > 1). At *L*_3_, PR has already been fully folded and little pulling force is generated (*F* ≈ 0, *f*_*FL*_ 0). (c) SDS-PAGE gel showing A and FL products for the *in vitro* translated, [^35^S]-Met labeled construct with *L* = 37 residues. Control constructs L37[A_c_] and L37[FLc] have, respectively, a stop codon and an inactivating Ala codon replacing the last Pro codon in the AP, leading to the production of exclusively *A*-sized and *FL*-sized proteins. Mw markers are indicated on the left.

Here, we have used a series of plasmid constructs to produce increasingly longer portions of the frameshifted HIV-1 TF-PR-RT precursor protein, with the C-terminal end of PR placed *L* residues upstream from the C-terminal end of the *E. coli* SecM (SecM(*Ec*)) AP, which in turn is followed by a 23-residue C-terminal tail [57], Figure 1a (see Supplemental Table S1 for amino acid sequences of all constructs). By varying *L*, PR can be positioned in different locations in the ribosome exit tunnel at the point when the ribosome reaches the last codon of the AP, and will therefore generate pulling forces of different magnitude depending on whether the peptide chain can fold or not in that location. In a construct where PR can start to fold at the point when the ribosome reaches the last codon of the AP, Figure 1b (middle), a strong pulling force *F* will be exerted on the nascent chain, which in turn reduces translational pausing at the AP and results in mostly full-length protein (including the C-terminal tail) being produced during a short pulse with [^35^S]-Met. In contrast, in constructs where PR is located too deep in the exit tunnel to be able to fold, or has already folded before the point when the ribosome reaches the end of the AP, little force is exerted on the AP, translational pausing will be efficient, and more of the arrested form of the protein will be produced during a short pulse with [^35^S]-Met, Figure 1b (left and right). After *in vitro* translation in the *E. coli*-derived PURE coupled transcription-translation system [58] for 15 min., full-length (FL) and arrested (A) protein species are separated on a SDS-PAGE gel and the fraction of full-length protein is calculated as *f_FL_* = *I_FL_*/(*I_FL_* + *I_A_*), where *I_FL_* and *I_A_* are the intensities of the bands representing FL and A species, respectively, Figure 1c. Because *f_FL_* is sensitive to pulling force, it can be used as a proxy for *F* [53,59–61]. A force profile (FP), in which *f_FL_* is plotted against *L* (i.e., each construct generates one data point), thus can be used to follow the cotranslational folding across the length of PR.

### PR undergoes a cotranslational folding event starting at L ≈ 25 residues

**Figure 2.**
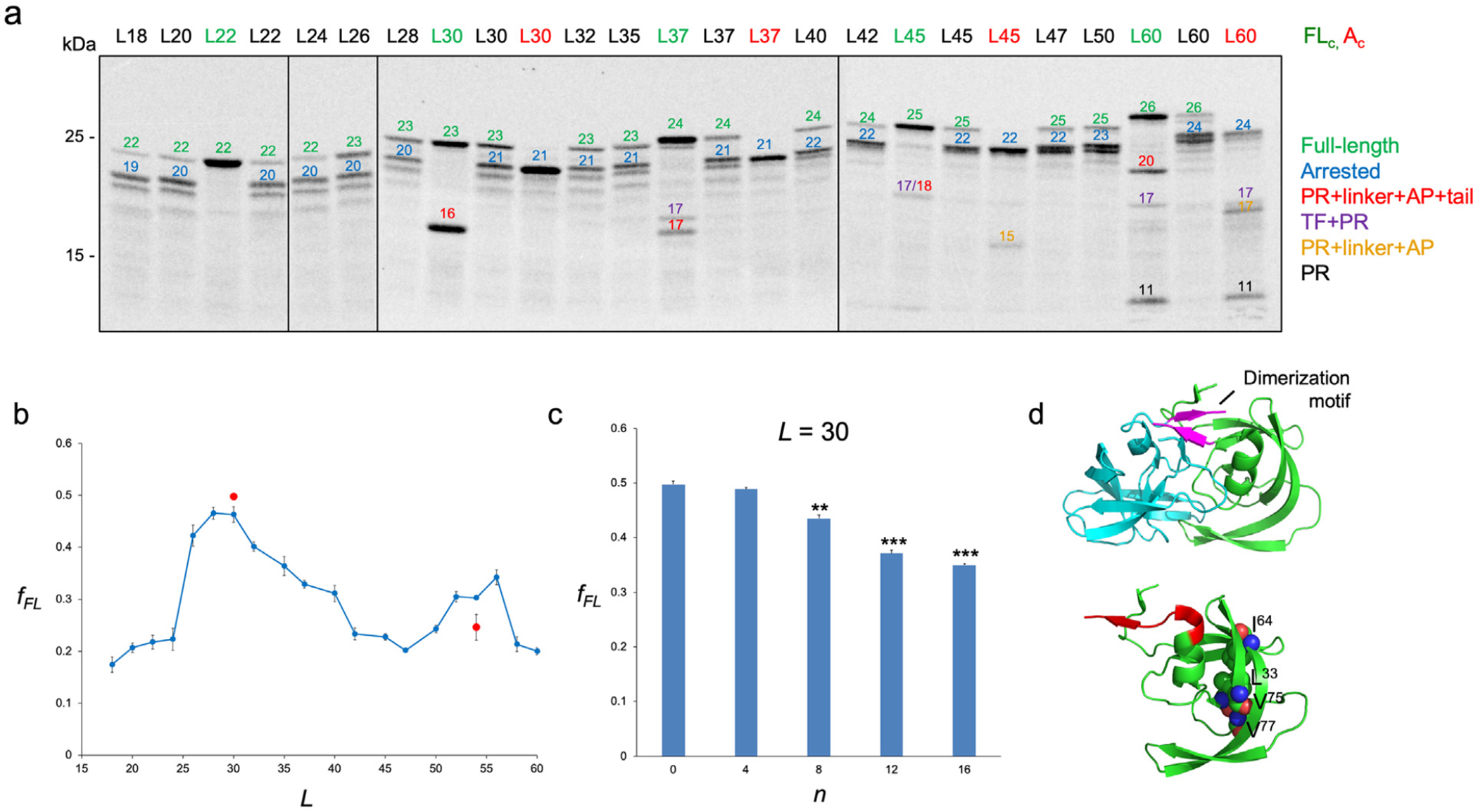
PR force profile. (a) 15 min. *in vitro* translations of constructs with an active AP (constructs indicated in black), FL_c_ controls (constructs indicated in green), and A_c_ controls (constructs indicated in red). Full-length, arrested, and proteolysis products are indicated by their calculated Mw’s (color-coded as indicated on the right). (b) FP for constructs with *L* = 18–60 residues (See Supplemental Table S1 for sequences). The red data points are for the quadruple PR mutant L^33^D + I^64^D + V^75^D + V^77^D. Each data point represents the average ± SEM of at least three replicates (*i.e.*, independent PURE *in vitro* translation reactions). (c) *f_FL_* values for the *L* = 30 construct with increasing numbers, *n*, of C-terminal residues replaced by alternating GS repeats (e.g., *n* = 4 means GSGS). Residues replaced in the *n* = 8 mutant are indicated in red in *d*, lower panel. (d) Upper panel: 3D structure of the PR dimer (PDB ID 4QJ6) with one monomer in cyan and the other in green. The C-terminal β-strands (residues T^96^–F^99^) are shown in magenta. Lower panel: one PR monomer showing the eight C-terminal residues Q^92^-F^99^ (in red) and the locations of the residues mutated in the quadruple PR mutant (in spacefill).

In order to detect cotranslational folding events in PR as it emerges from the ribosome exit tunnel, a FP was determined using a series of constructs where *L* was varied between 18 and 60 residues (named L18–L60), Figure 2a (see Supplemental Table S1 for sequences); for some *L* values we also made control constructs in which the AP was inactivated either by mutating its C-terminal Pro to Ala (FL_c_ controls, producing only full-length protein), or by mutating its C-terminal Pro codon to a stop codon (A_c_ controls, producing only arrested-size protein), c.f., Figure 1c. Note that constructs with *L* ≤ 24 residues have short C-terminal deletions in PR, Supplemental Table S1.

As seen in Figure 2b, *f_FL_* increases sharply between *L* = 24 and *L* = 26 residues, is maximal at *L* = 28–30 residues, and returns to baseline at *L* = 42 residues, indicating a cotranslational folding event. A second peak of lower amplitude is seen for *L* = 50–56 residues. The maximal amplitude at *L* = 28–30 residues (*f_FL_* ≈ 0.5) is lower than what is typically seen for small, independently folding protein domains (which generate peaks with *f_FL_* ≈ 0.7–0.9 [49,62]), suggesting the formation of a folding intermediate of relatively low stability.

To probe the nature of the folding events causing the two peaks, we first mutated the L30 construct by replacing an increasing number of C-terminal PR residues with Gly-Ser (GS) repeats. This led to a significant decrease in *f_FL_* values when 8 or more residues were replaced, Figure 2c. Replacement of the C-terminal β-strand (T^95^LNF^99^) with GSGS thus has no effect on *f_FL_* in the L30 construct, but extending the replacements into the C-terminal α-helix (residues 86–94; Figure 2d) reduces *f_FL_*. We also simultaneously mutated four residues (L^33^, I^64^, V^75^, V^77^; Figure 2d) in the hydrophobic core of the PR dimer structure to D in the L30 and L54 constructs. This had no effect on *f_FL_* for the L30 construct, but significantly reduced *f_FL_* for the L54 construct, Figure 2b (red data points).

We conclude that the first peak in the FP is caused by a cotranslational folding event that generates a relatively modest increase in *f_FL_* values and initiates when the C-terminal end of PR reaches ~25 residues from the PTC. This folding event does not seem to involve the entire PR monomer, but appears to be caused mainly by the formation of the C-terminal α-helix which at that point has its C-terminal end located ~30 residues from the PTC, within the vestibule of the exit tunnel; the major part of PR thus emerges from the exit tunnel in an unfolded, possibly collapsed, state. The folded form of a monomeric PR mutant lacking the dimerization-mediating, short C-terminal β-strand (T^95^LNF^99^) is only marginally stable [41,63], and the monomer may be further destabilized when held in proximity to the ribosome [64–66], possibly explaining the absence of a folding event involving the whole PR domain.

The second peak, at *L* = 50–56 residues, corresponds to a situation where the entire PR domain is exposed outside the exit tunnel, as only ~35–40 residues of an extended nascent chain are embedded within the ~100 Å long tunnel [67,68]. At these chain lengths, it is possible that the PR segment on the arrested ribosome can reach and dimerize with the PR segment on the ribosome stacked behind it on the mRNA, or with an already synthesized and released TF-PR-RT chain [69], causing the observed increase in *f_FL_*. A dimerization event would be consistent with the observed effect on *f_FL_* of the hydrophobic core mutations in the L54 construct, although we cannot rule out other causes for this peak. Taken together, the FP data suggests that in the infected cell, the PR monomer folds into a marginally stable structure while still attached to the ribosome.

### In vitro-translated TF-PR-RT constructs produce active PR

As noted above, while measuring the FP, we also expressed control constructs with mutant arrest peptides which do not arrest but are released normally as either full-length (FL_c_) or arrested-size (A_c_) products. As seen in Figure 2a, various cleavage products were observed for the *L* ≥ 30 residues FL_c_ constructs and for the *L* ≥ 45 residues A_c_ constructs, suggesting the formation of active PR; notably, however, no such cleavage products were seen during the 15 min. translation reaction for any of the constructs with an active AP that are retained on the ribosome. These observations piqued our interest, and we decided to look more deeply into the processing of the TF-PR-RT constructs.

Constructs with *L* ≤ 24 residues do not contain the C-terminal β-strand (T^95^LNF^99^) required for dimerization of PR [40], and hence are not expected to produce active enzyme. Moreover, while all constructs contain the cleavage site SFSF/PQIT between TF and PR (see Figure 1a), only constructs with *L* ≥ 34 residues contain the native cleavage site TLNF/PISP between PR and RT (Supplemental Table S1). Consistent with this, for L30[FL_c_] we see only one cleavage product with a Mw corresponding to that expected for the PR + linker + AP + tail fragment (16.4 kDa; Figure 2a) representing cleavage between TF and PR, while for L37[FL_c_] we see two cleavage products of Mw ~ 17 kDa, close to the expected 17.2 kDa for the PR + linker + AP + tail fragment and 17.3 kDa for the TF + PR fragment (*c.f.*, Figure 1c), representing products cleaved at either the N-terminus or the C-terminus of PR. In addition, for L60[FL_c_] there is a band at ~11 kDa which is approximately the Mw of mature PR (10.8 kDa), *i.e.*, cleavage at both ends of PR in the same PR precursor. In contrast, there are no cleavage products seen for L30 [A_c_] and L37[A_c_], but a fragment with the size expected for a PR + linker + AP product (15.5 kDa) is seen for L45[A_c_], while two bands at ~ 17 kDa, close to the Mw’s expected for the TF + PR and PR + linker + AP products, and one band at the Mw expected for PR, are seen for L60 [A_c_]. The expected TF fragment has a calculated Mw of 6.5 kDa and is too small to be visible on the gels.

**Figure 3.**
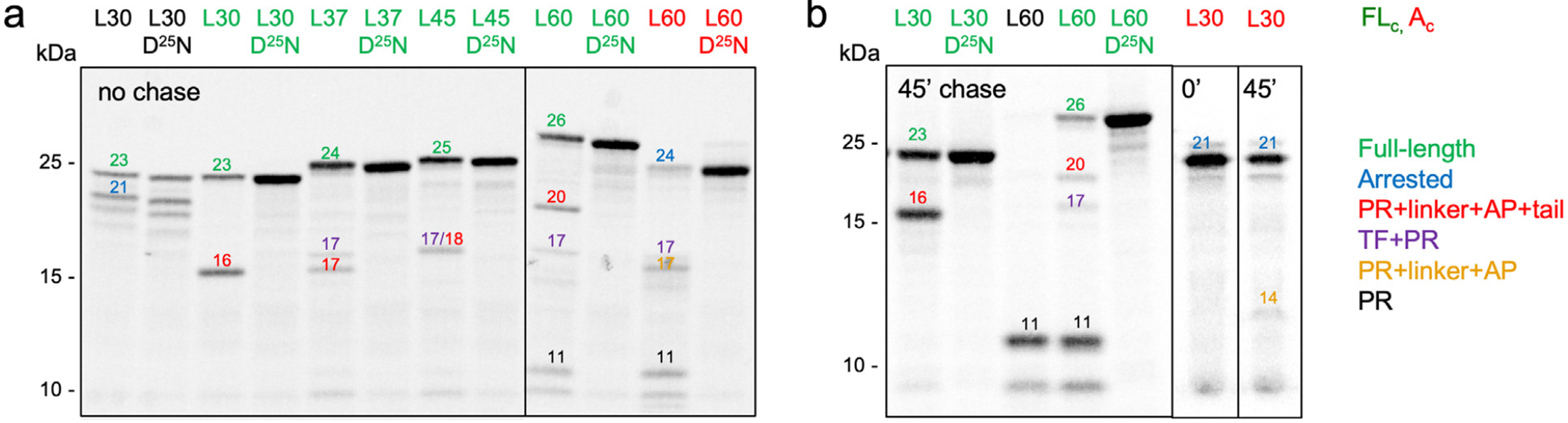
Autoproteolytic cleavage of TF-PR-RT constructs. (a) SDS-PAGE gels of *in vitro* translated, [^35^S]-Met labeled constructs (indicated in black), together with A_c_ (indicated in red) and FL_c_ (indicated in green) control constructs. Cleavage products are identified by their Mw’s according to the color code on the right. (b) Same as in panel *a*, but after a 45 min. chase with excess non-radioactive Met. The ~9.5 kDa fragment at the bottom of the gels is a contamination from the PURE system itself, see Figure 6a. The L30[A_c_] lanes in panel *b* are also shown in Figure 6b.

To confirm that the observed fragments are produced by PR-mediated cleavage of the FL_c_ and A_c_ products, we repeated the experiments with the inactivating PR(D^25^N) mutation [70]. As seen in Figure 3a, no cleavage products were seen for the tested constructs in this case. Thus, the *in vitro* translation reactions produce active PR, at least for the FL_c_ and the longer A_c_ constructs. The failure to cleave shorter A_c_ constructs is not due to inaccessibility of the cleavage site. The TF/PR cleavage site in L30[A_c_], which is not cleaved, is 129 amino acids from the C-terminus; in contrast the PR/RT cleavage site in L37[FL_c_], which is cleaved, is only 60 amino acids from the C-terminus. Thus, it is the ability to form active dimeric protease that is the determinant of cleavage for constructs that include the PR C-terminal β-strand.

### Ribosome-attached nascent TF-PR-RT chains are susceptible to cleavage by PR but released polyprotein is not

In the following experiments we explore both formation of active protease and states when the protease precursor protein can be cleaved as protease substrate. To preview these experiments, the data show that the protease precursor must be bound to the ribosome (a “constrained monomer”) to be a substrate, that uncleaved precursors that are released from the ribosome and dimerize are refractory to cleavage by the protease, and that the initial dimerization event to give active protease involves dimerization of precursors still attached to the ribosome.

To analyze the cleavage reactions in more detail, we performed an initial set of pulse-chase experiments on L30[FL_c_], L30(D^25^N)[FL_c_], L30[A_c_], L60, L60[FL_c_], and L60(D^25^N)[FL_c_]. The pulse was done for 15 min in the presence of [^35^S]-Met with an aliquot taken at the end of the pulse (Figure 3a, no chase or 0′). Non-radioactive Met was added in ~15,000-fold excess after the 15 min. pulse incubation, and samples were collected after an additional 45 min. chase (Figure 3b). The relative amounts of cleavage products remained approximately the same for L30[FL_c_] and L60[FL_c_] before and after the chase, Figure 3a, b. In marked contrast, L30[A_c_] was largely refractory to autoproteolysis, and only a small amount of processed PR + linker + AP was seen after the chase, Figure 3b (right panel). Thus, L30[A_c_] produces only marginal amounts of catalytically active protease during the 45 min. chase, whereas the closely related L30[FL_c_] construct that includes the 23-residue C-terminal tail produces ample amounts of active protease even without a chase. The most obvious difference between these two constructs is that the entire TF-PR segment (located 53 residues from the stop codon in L30[FL_c_]) is exposed outside the ribosome exit tunnel for a time corresponding to the translation of ~10–15 3′ codons (*i.e.*, ~10–20 s [71]) before synthesis of the L30[FL_c_] chain terminates and it is released from the ribosome, while the C-terminal β-strand in PR (located 29 residues from the end of L30 [A_c_]) does not appear outside the exit tunnel at any point before chain termination of L30[A_c_]. This suggests that the initial TF-PR dimerization reaction – the first step in the production of active PR – involves at least one ribosome-bound nascent chain.

The ribosome-arrested L60 construct, which did not show any cleavage products after a 15 min. translation reaction (Figure 2a), was efficiently converted to mature ~11 kDa PR during the chase, Figure 3b, unless the PR inhibitor darunavir [72] was included in the reaction (Supplemental Figure S1). Furthermore, L60 undergoes complete autoproteolysis to PR, showing none of the intermediate cleavage products observed in the non-arrested L60[FL_c_] control, Figure 3b. This suggests that ribosome-bound nascent chains are the main PR substrates and that for L60 both cleavage sites flanking PR are well exposed while anchored on the ribosome.

**Figure 4.**
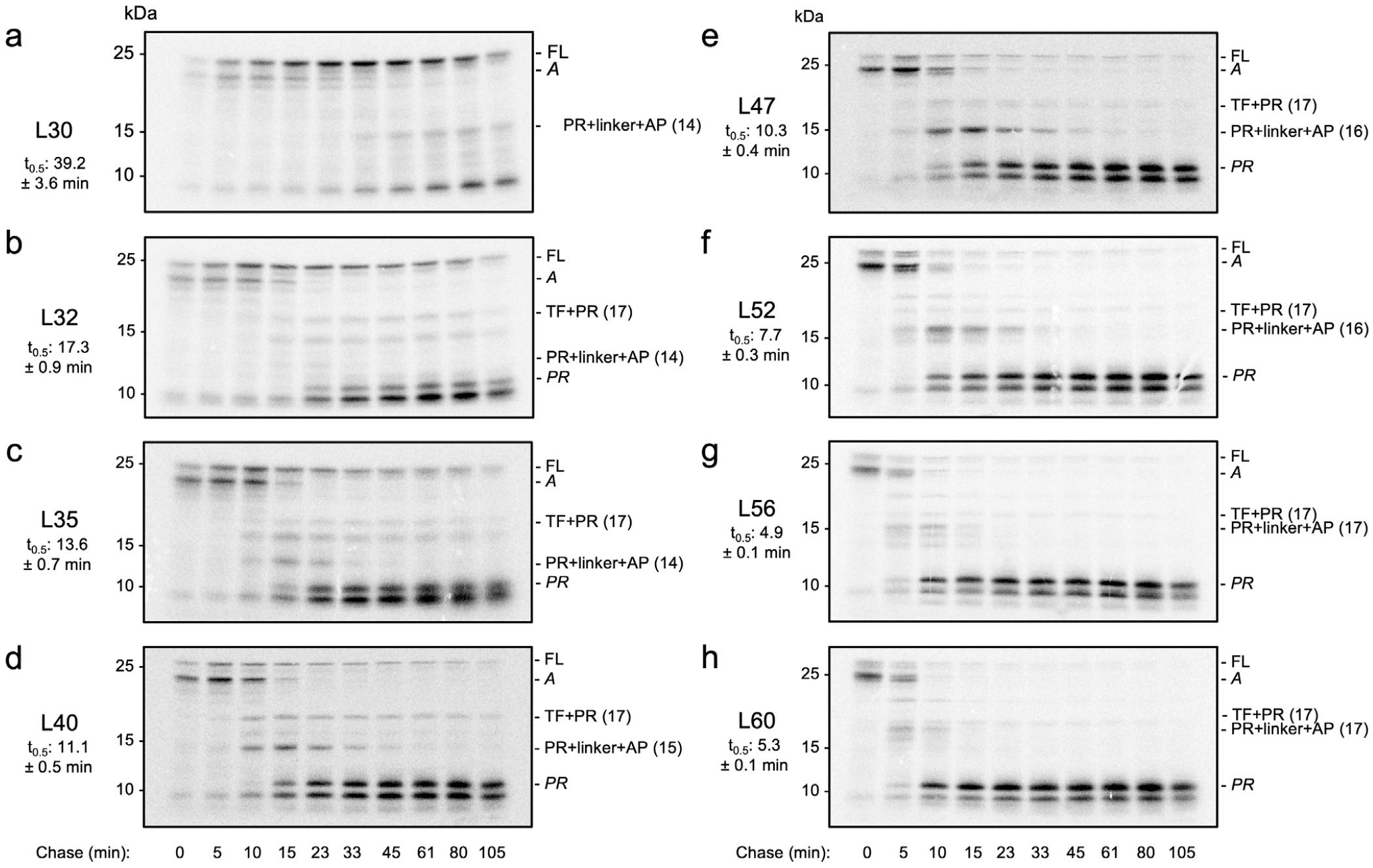
Pulse-chase analysis of the autoproteolytic cleavage reaction. (a) Constructs were translated *in vitro* for 15 min. in the presence of [^35^S]-Met and then chased after addition of excess non-radioactive Met for the indicated times. Full-length (FL), arrested (A), ~11 kDa mature PR, TF + PR, and variable-length PR + linker + AP products are indicated (with calculated Mw’s). L30 is missing the PR/RT cleavage site, and therefore cannot generate mature PR. Lag-time midpoints (*t*_0.5_) ± the standard error of the fitting are indicated. See Methods and Supplemental Figure S2 for details.

A more extensive pulse-chase analysis of a range of ribosome-arrested constructs (*L* = 30–60 residues), Figure 4, showed typical autoproteolytic cleavage kinetics, with a slow initial lag phase during which the first, catalytically active PR molecules are produced (presumably via dimerization of a weakly active TF-PR-RT precursor to generate more active forms of PR dimers), followed by rapid processing of the arrested (but not full-length) form, first to the ribosome-bound PR + linker + AP fragment, and then to fully cleaved ~11 kDa PR (see Supplemental Figure S2 for quantitative analysis). As expected from the absence of an intact PR/RT cleavage site in the L30 construct, no ~11 kDa PR was produced from L30 during the 45 min. chase (Figure 4a), although the arrested form slowly converts to a ~14 kDa fragment (lag-time midpoint *t*_0.5_ = 39 min.), which may correspond to the 14.0 kDa PR + linker + AP fragment [53]. Notably, autoproteolytic cleavages are seen only for the arrested (A), ribosome-bound forms of the proteins, not for the released full-length (FL) products which are largely stable during the chase period and refractory to cleavage.

In agreement with the hypothesis that at least one ribosome-bound nascent chain is required for the initial TF-PR dimerization reaction, longer constructs in which arrested TF-PR-RT nascent chains are more exposed outside the ribosome and hence better available for dimerization were more rapidly processed to mature ~11 kDa PR, Supplemental Figure S3a. Restoration of the PR/RT cleavage site (TLNF/PISG; same as in the L32 construct) made the arrested form of the L30 construct susceptible to autoproteolytic processing to mature PR, but with markedly slower kinetics than seen for the L32 construct, Supplemental Figure S3b.

**Figure 5.**
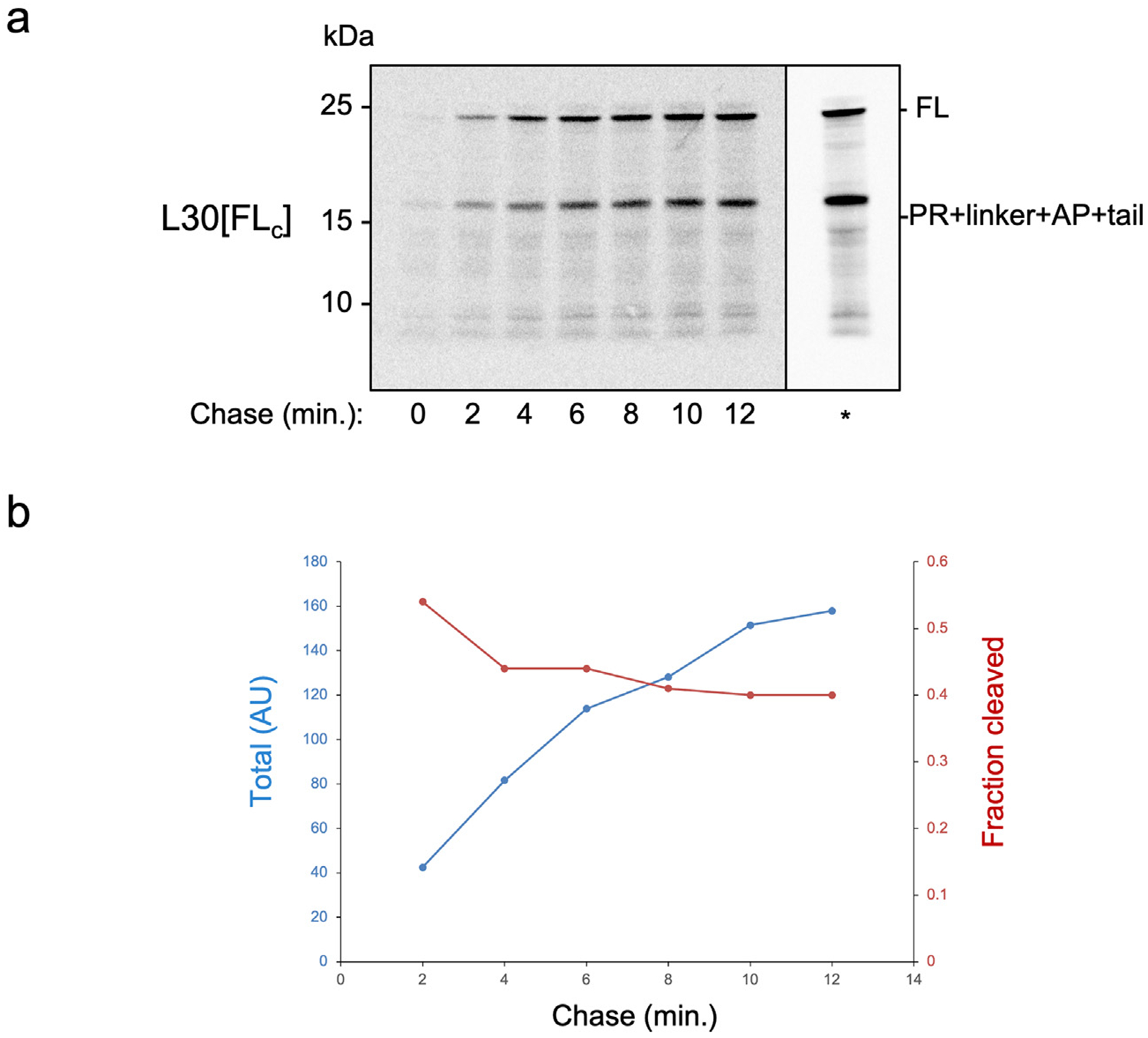
Rapid pulse-chase analysis of the L30[FL_c_] construct. (a) PURE *in vitro* translation reactions were carried out for 2 min. in the presence of [^35^S]-Met followed by a chase in the presence of excess non-radioactive Met. The lane indicated by * is for a 15 min. translation reaction in the presence of [^35^S]-Met with no chase, included for comparison. (b) Quantitation of the data in panel *a*, showing the total intensity of the 23 kDa + 16 kDa bands (arbitrary units, blue curve) and the fraction cleaved product (red curve).

Rapid (2 min. labeling) pulse-chase analysis of the autoproteolysis reaction for the non-arrested L30[FL_c_] construct revealed a very different kinetic behavior, Figure 5. In this case, the PR + linker + AP + tail cleavage product appeared together with the full-length chains already after a ~2 min. chase, *i.e.*, as soon as the 210-residue long full-length chains were produced (the translation rate in the PURE system is ≲1 aa/s [71,73]), and the relative amounts of cleaved *vs*. full-length products remained approximately constant during the chase (*c.f.*, Figure 3a, b). Thus, there is no detectable delay in the production of active PR for non-arrested constructs; nevertheless, there is only partial processing of the full-length chains. Again, this suggests that only ribosome-attached nascent chains are accessible to proteolysis, while released full-length or partially processed chains are not.

**Figure 6.**
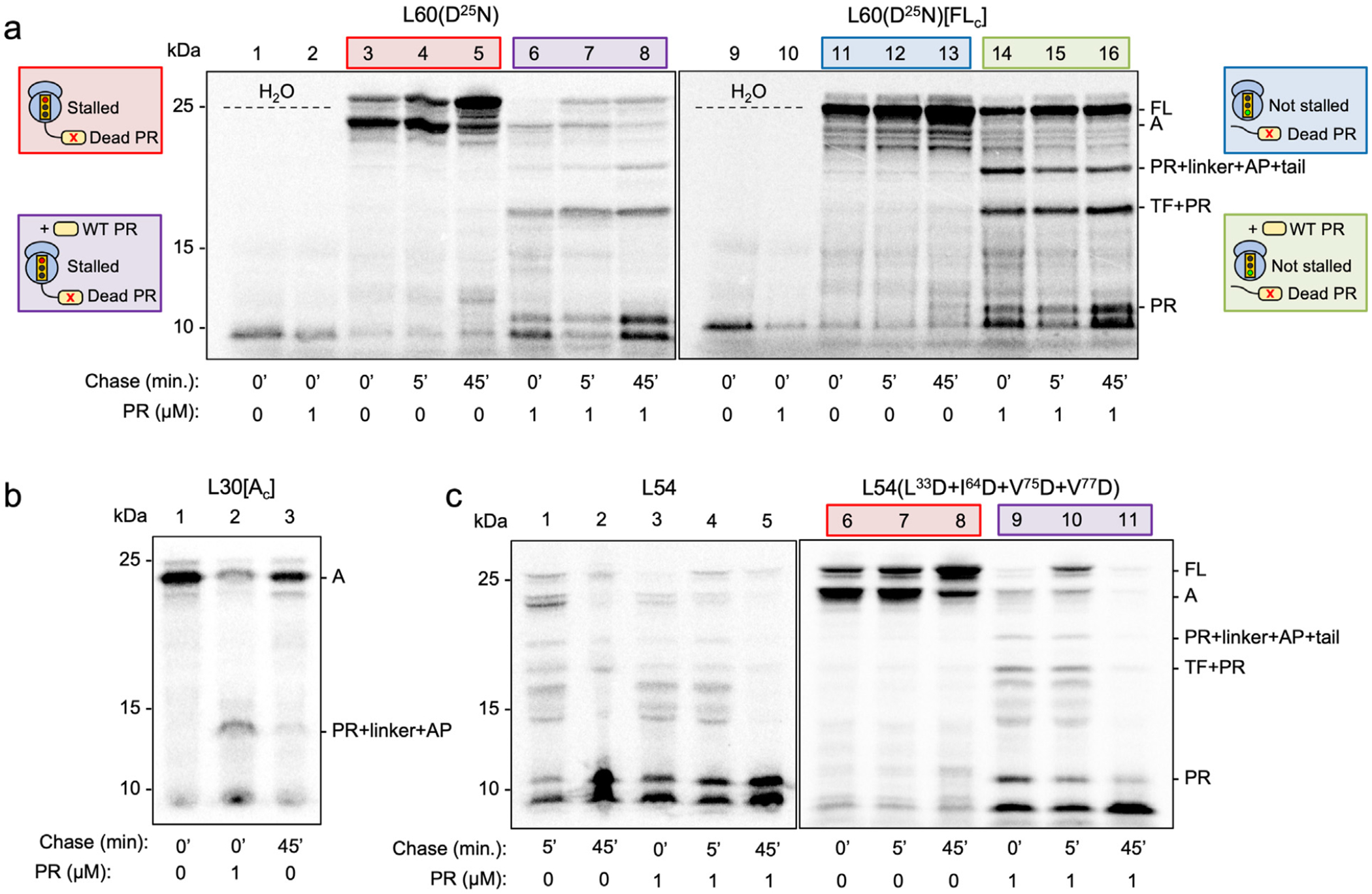
“Spiking” of the in vitro translation reaction with active PR. (a) Lanes 1–8: Inactive L60(D^25^N) was translated *in vitro* for 15 min. in the presence of [^35^S]-Met, followed by a chase in the presence of an excess of non-radioactive Met. Reactions were carried out in the presence or absence of purified, active PR at 1 μM final concentration, as indicated. PR was added at the beginning of the chase, except in reactions with no chase, where it was added at the beginning of the reaction. The first two lanes, marked *H_2_O*, were reactions carried out without added construct DNA. Lanes 9–16: Same as in lanes 1–8, but for L60(D^25^N)[FL_c_]. (b) Same as in panel *a*, but for L30[A*c*]. (c) Same as in panel *a*, but for L54 (lanes 1–5) and L54(L^33^D + I^64^D + V^75^D + V^77^D) (lanes 6–11).

To separate the effects of the initial, dimerization-dependent production of PR from the PR-mediated cleavage of nascent TF-PR-RT chains, we performed a ‘spiking’ experiment on the L30[A_c_], L60(D^25^N), and L60(D^25^N)[FL_c_] constructs by adding purified active PR (1 μM final concentration) to the translation reaction, either at the start of the 15 min. [^35^S]-Met labeling period, or together with the non-radioactive Met at the start of the chase period. For the ribosome-arrested L60(D^25^N) construct, Figure 6a (lanes 1–8), inclusion of PR during the labeling period led to efficient reduction in both the FL and A products, with a concomitant appearance of both partial (PR + linker + AP + tail, TF + PR) and full (PR) cleavage products (compare lanes 3 and 6); a similar cleavage profile was seen when active PR was added at the start of the chase period, except that the FL product was now largely protease-resistant (compare lanes 4 and 7) and did not decrease in intensity during the 45 min. chase (compare lanes 7 and 8). Similar results, although with significantly lower levels of proteolysis, were seen for the non-arrested L60(D^25^N)[FL_c_] construct (lanes 9–16): appearance of partial and full proteolysis products at early time points (lanes 14, 15), but no further processing during the 45 min. chase (lane 16), again demonstrating that both the FL and partial proteolysis products are largely refractory to proteolysis after they have been released from the ribosome. The 9.5 kDa band seen also in previous Figures appears even when no DNA was added to the PURE reaction (lanes 1, 2 and 9, 10), hence this band originates from the PURE system itself. For L30[A_c_] – which produces only marginal amounts of cleaved product even during a 45 min. chase, Figure 3 – ‘spiking’ with purified PR led to a rapid appearance of the PR + linker + AP product, Figure 6b, amounting to ~50% of the total. Thus, ribosome-bound L30[A_c_] is a good substrate for PR but is very inefficient in forming the initial TF-PR dimers.

We also tested L54 and the presumably non-dimerizing, inactive mutant L54(L^33^D + I^64^D + V^75^D + V^77^D) in the spiking assay, Figure 6c. L54 behaves as expected, *i.e.*, both the FL and A products are largely proteolyzed to the ~11 kDa product when PR is present during the labeling period (lane 3), whereas the FL product remains largely PR-resistant when PR is added at the start of the chase period (compare lanes 1 and 4, and lanes 2 and 5). In contrast, for L54 (L^33^D + I^64^D + V^75^D + V^77^D), the FL product is sensitive to PR during the chase, and little FL remains after 45′ chase in the presence of PR (compare lanes 8 and 11).

As a control, we subjected protease-dead D^25^N variants of a few constructs to pulse-proteolysis [66] by thermolysin, a promiscuous protease with little sequence specificity. In this experiment, constructs were translated for 15 min. in the PURE system, and then briefly incubated for 1 min. with thermolysin. As seen in Supplemental Figure S4a, both the full-length and arrested forms of L30 (D^25^N), L60(D^25^N), L60(D^25^N)[FL_c_], and the arrest-enhanced L60(D^25^N)-3W construct (see next section) were efficiently cleaved by thermolysin, and all yielded a PR-sized ~11 kDa fragment (though barely visible for L30(D^25^N)). We also tested L54 and the non-dimerizing hydrophobic core mutant L54(L^33^D + I^64^D + V^75^D + V^77^D), Supplemental Figure S4b. As expected, L54 yielded PR-sized ~ 11 k Da product, whereas L54(L^33^D + I^64^D + V^75^D + V^77^D) yielded no ~11 kDa product. The simplest interpretation of these data is that the PR-portion of released full-length products forms a PR- and thermolysin-resistant dimer (except in the non-dimerizing mutant), while the TF and RT portions remain flexible and susceptible to degradation by thermolysin.

### The lag-time midpoint increases with the strength of the AP

**Figure 7.**
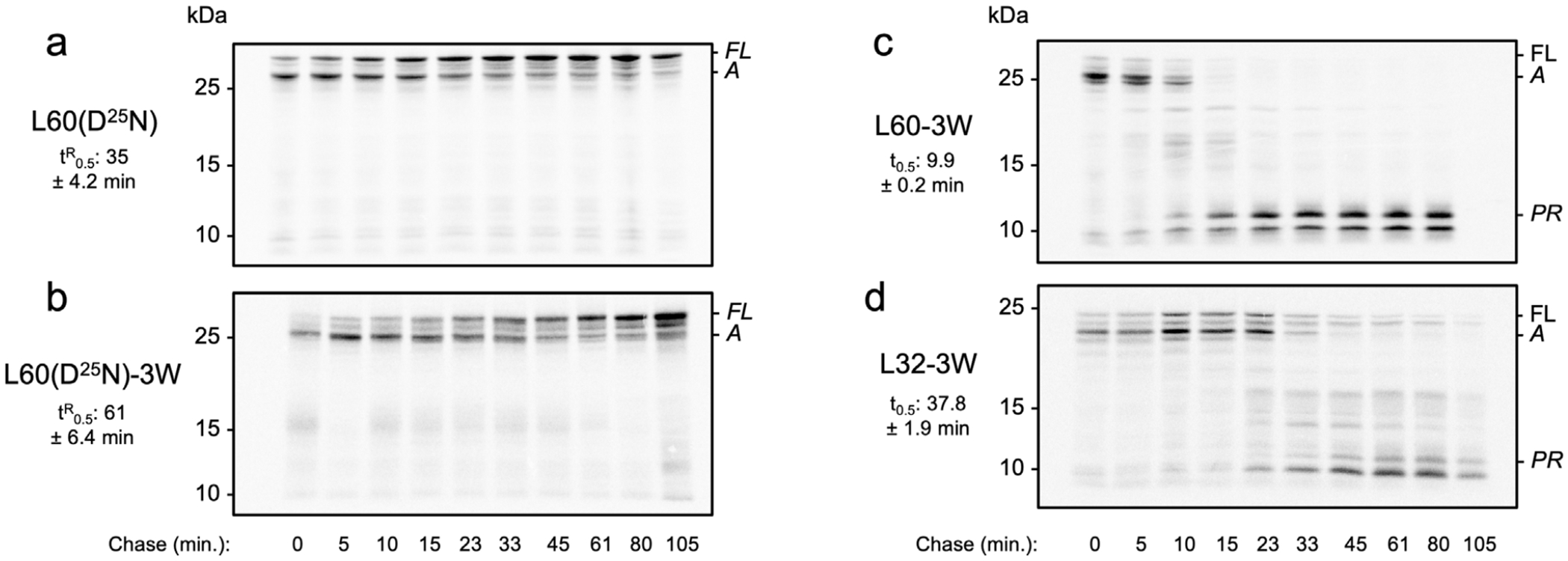
Pulse-chase analysis of SecM(Ec)-3W AP constructs. Pulse chase experiments were performed as in Figure 4. FL, A, and PR products are indicated. *3 W* indicates that the strong SecM(*Ec*)-3W mutant AP was used. Release-time midpoints ($\left(t_{0.5}^{R}\right)$) are indicated in panels *a* and *b*, and lag-time midpoints in panels *c* and *d*. (see Supplemental Figure S2 for quantitations).

In a final set of experiments, we tested the effects of the AP itself on the autoproteolytic reaction, comparing the SecM(*Ec*) AP to the much stronger SecM(*Ec*)-3W AP [54]. As expected from the extent of translational pausing caused by the SecM(*Ec*) AP at low pulling forces [53], a slow conversion of the arrested to the full-length form (release-time midpoint $t_{0.5}^{R}=35\;\text{min}$) was seen for the catalytically inactive L60(D^25^N) mutant, Figure 7a (also evident in Figure 6a). Using the stronger SecM(*Ec*)-3W AP that retains the nascent chain on the ribosome more efficiently than does the wild-type SecM(*Ec*) AP, the conversion of the arrested to the full-length form of L60(D^25^N)-3W took even longer ($t_{0.5}^{R}=61\;\text{min}$), Figure 7b.

With the stronger SecM(*Ec*)-3W AP, the long L60 construct in which the entire PR domain is exposed outside the ribosome in the arrested nascent chain still underwent rapid autoproteolytic processing but with a two-fold increase in the lag-time midpoint (*t*_0.5_ = 9.9 min. *vs*. 5.3 min. with the SecM(*Ec*) AP), Figure 7c, which parallels the ~2-fold increase in the release-time midpoint between the SecM(*Ec*) and the SecM(*Ec*)-3W APs. Autoproteolysis of the short L32 construct in which the C-terminal end of the PR domain is sequestered inside the exit tunnel in the arrested nascent chain was also delayed by the SecM(*Ec*)-3W AP, again with a ~2 fold increase in *t*_0.5_ from 17 min. for the SecM(*Ec*) AP to 38 min. for the SecM(*Ec*)-3W AP, Figure 7d. Thus, the release-time midpoint appears to set the rate at which active PR is produced, implying that the initially formed TF-PR-RT dimers must be released from the ribosome in order to start cleaving other ribosome-bound TF-PR-RT nascent chains.

## Discussion

We have analyzed the cotranslational folding and proteolysis of frameshifted TF-PR-RT constructs of different lengths using both FPA, autoproteolysis, and pulse-proteolysis assays in the *E. coli*-derived PURE *in vitro* transcription-translation system. In short, we find that the PR monomer starts to fold in the ribosome exit tunnel when the end of its C-terminal helix is ~30 residues away from the PTC (Figure 2b). Previous studies suggest that at this depth the exit tunnel can accommodate folding of protein domains of up to ~45 amino acids long, whereas domains the size of the PR monomer (~99 amino acids) are able to fold only when ≳ 35 residues away from the PTC [62]. Hence, the observed folding event is unlikely to involve the whole monomer, and in fact is not affected when core hydrophobic residues in folded PR are mutated, Figure 2b. However, mutations in the C-terminal helix reduce the amplitude of the peak in the FP at *L* = 30 residues (Figure 2c), suggesting that a main contribution to the peak is from folding of the C-terminal helix.

A second peak of lower amplitude is seen at *L* = 52–56 residues, at *L* values too large to represent the folding of the PR monomer [62]. At such large *L* values, the PR part of the TF-PR-RT nascent chain should be sufficiently far out of the ribosome to be able to dimerize with another TF-PR-RT molecule [74], either in *cis* (*i.e.*, with TF-PR-RT being synthesized on the same mRNA by the trailing ribosome) or in *trans* (*i.e.*, with TF-PR-RT being synthesized on a different mRNA, or with released full-length TF-PR-RT). Both types of cotranslational dimerization have been described [69,74]. As expected for a dimerization event, mutations in the hydrophobic core of PR reduce the amplitude of this peak (Figure 2b).

We have also found that catalytically active PR dimers can form autoproteolytically from the TF-PR-RT protein in the PURE *in vitro* transcription/translation system, as has been shown before for the rabbit reticulocyte *in vitro* translation system [34]. Unexpectedly, however, processing of the TF-PR-RT protein is seen only for arrested, ribosome-attached chains, while released full-length and partially processed chains remain uncleaved even after a 105 min. chase (Figure 4). Similarly, as seen for the L60(D^25^N) and L60 (D^25^N)[FL_c_] constructs, full-length and proteolysis products not bound to the ribosome are largely resistant to purified, active PR added to the translation reaction, except for the non-dimerizing L54(L^33^D + I^64^D + V^75^D + V^77^D) mutant (Figure 6). Thus, PR cleaves TF-PR-RT while it is constrained as a monomer on the ribosome but not after its release from the ribosome, presumably because of post-release dimerization of the PR portion that makes the TF/PR and PR/RT cleavage sites inaccessible to PR. Consistent with this interpretation, the promiscuous thermolysin protease readily degrades both ribosome-bound and released TF-PR-RT to a PR-sized fragment (Figure S4).

Our results further suggest that the initial dimerization of TF-PR-RT monomers involves at least one ribosome-bound nascent chain. Thus, L30[FL_c_] produces ample amounts of active PR already after a 2 min. translation reaction followed by a 2 min. chase (Figure 5), while the closely related L30[A_c_] produces only trace amounts of active PR even after a 15 min. translation reaction followed by a 45 min. chase (Figure 3b). The only difference between these two constructs is the 23-residue C-terminal tail present in L30[FL_c_], which causes the TF-PR portion to be exposed outside the ribosome exit tunnel for ~10–15 s before chain termination in L30[FL_c_], but not in L30[A_c_].

In contrast to FL_c_ and A_c_ controls, arrested nascent chains spend a considerable time in the ribosome-bound state before being released, either by slow release of the AP or by autoproteolytic cleavage at the PR/RT cleavage site. As seen by comparing the initial lag-time midpoint in the onset of autocatalytic processing (*t*_0.5_) between constructs arrested by the medium-strong SecM(*Ec*) AP and the very strong SecM(*Ec*)-3W AP (Figure 7), *t*_0.5_ appears to be set by the release-time midpoint ($t_{0.5}^{R}$), again implying that the rate-limiting step in the production of active PR for constructs with an active AP is the release of TR-PR-RT dimers from the ribosome.

We have not explored the form(s) of active protease in this system. Since the full-length precursor is the initial product, this form must have some protease activity. However, as seen with L30[A_c_], in the absence of cleavage at either boundary of the PR this form of the precursor is only weakly active (Figure 3b). In contrast, activity is easily detected in the L30[FL_c_] construct where the TF/PR site is cleaved. The adjacent TF region has been shown to negatively impact PR activity [38,43,44]. It seems likely that cleavage at the TF/PR site creates processing intermediates that as either homodimers or heterodimers with uncleaved precursors have increased activity to carry out the observed early processing events.

**Figure 8.**
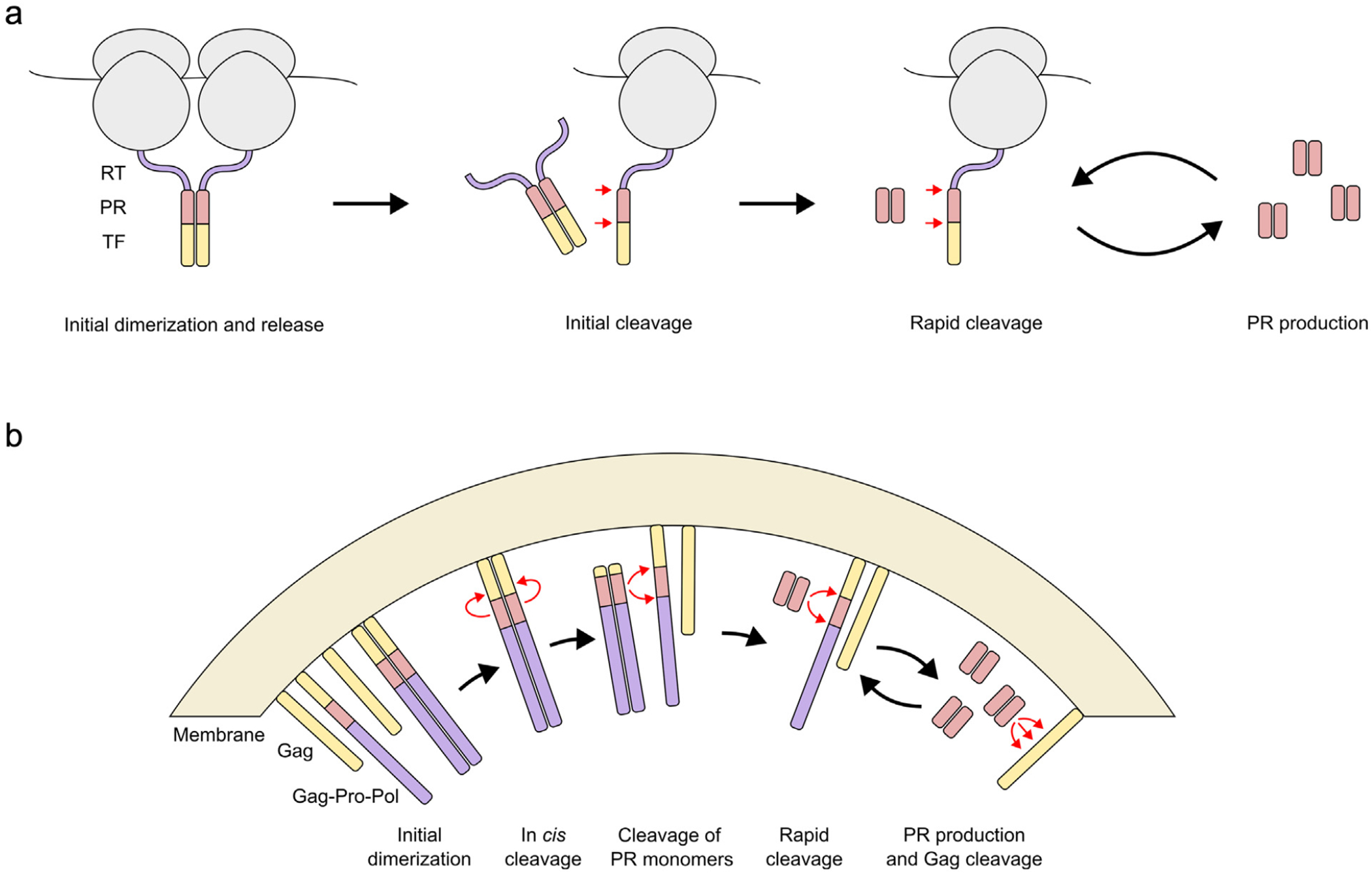
Models for autocatalytic processing of TF-PR-RT constructs. (a) Cotranslational processing during *in vitro* translation (this work). (b) Protease activation during virion assembly.

As illustrated in Figure 8a, under the *in vitro* conditions employed here, the TF-PR-RT protein can, to a first approximation, engage in the initial dimerization reaction required to produce a weakly active PR, but the TF-PR-RT protein is susceptible to PR-mediated cleavage only when still attached to the ribosome as a monomer. Released full-length or partially processed chains form low-activity TF-PR-RT dimers that are not in themselves substrates for PR, and hence remain unprocessed. As both the TF/PR and PR/RT cleavage sites are sequestered in a four-stranded β-sheet in the PR dimer (Figure 2d) they are presumably not available for processing in folded TF-PR-RT dimers. This model does not require the initial TF-PR-RT dimer to self-cleave [44–48] in order for the first active PR molecules to be released, because monomeric, ribosome-bound nascent monomeric TF-PR-RT substrates will be available on neighboring ribosomes.

In an infected cell, Gag and Gag-Pro-Pol are transported intact to the sites of virion assembly post-translation; however, there are elements of the *in vitro* translation system in terms of constrained PR monomers and the initial release of low activity longer dimers that may mimic virion assembly and maturation. In the HIV-1 infected cell, cotranslational dimerization of the PR domain is largely precluded by the low frequency (−5%) of the −1 frameshifting required to produce Gag-Pro-Pol, as only 1 in ~400 ribosomes would both carry nascent Gag-Pro-Pol and have a neighboring ribosome also carrying Gag-Pro-Pol that can dimerize; thus the Gag-Pro-Pol precursor will transit to the site of virion assembly either as a monomer or, if low order complexes form, in complex with Gag but not Gag-Pro-Pol. It is during virion assembly when PR monomers constrained in Gag-Pro-Pol monomers may behave as the constrained monomers we have studied when retained on ribosomes in the *in vitro* translation system.

There are approximately 2500 Gag molecules and thus 125 Gag-Pro-Pol molecules anchored on the inner face of the viral membrane in a budding virion [2]. If Gag-Pro-Pol molecules enter the budding particle as monomers (or as low complexity oligomers with Gag), then the chance juxtaposition of two Gag-Pro-Pol molecules during assembly, possibly potentiated by the curvature of budding, would allow an infrequent dimerization event (at least one per virion on average). Such a dimerization event would be followed by upstream cleavage at SP2/NC and in TF in *cis* [34] to release a TF-PR-RT-IN precursor dimer from the viral membrane. This dimeric form of the precursor could then diffuse within the lumen of the virion to effect cleavage of monomeric Gag-Pro-Pol that would then generate PR monomers. The PR monomers would form the highly active dimers needed to carry out all of the cleavage events needed for virion maturation, Figure 8b.

## Materials and Methods

### Enzymes and chemicals

Enzymes and other reagents were purchased from Thermo Fisher Scientific (Waltham, Massachusetts, U.S.), New England BioLabs (Ipswich, Massachusetts, U.S.), and Sigma-Aldrich (subsidiary of Merck Life Science, Darmstadt, Germany). Oligonucleotides were ordered from Eurofins Genomics (Luxembourg City, Luxembourg) and Thermo Fisher Scientific. L-[^35^S]-methionine (NEG009T001MC) was provided by PerkinElmer (Shelton, Connecticut, U.S.).

### Cloning and mutagenesis

The DNA sequence of Gag-Pro-Pol was acquired from the pNL4-3 clone of HIV-1 [75]. To focus on PR and its immediate fusion neighbors, we used a synthetic gene that begins with TF already in the −1 frame and ends at RT residue 30. A variable-length linker, the 17-residue SecM(*Ec*) arrest peptide, and a 23-residue C-terminal tail were added to the C-terminus, Figure 1a, and the resulting ORF was subcloned downstream of a T7 promoter in a pET-19b vector carrying an ampicillin resistance cassette. Simple mutagenesis was performed by mismatch PCR, whereas more extensive mutagenesis was performed with a combination of PCR and Gibson assembly [76]. See Supplemental Table S1 for amino acid sequences of all constructs.

### In vitro pulse-labeling analysis

Before expression, linear DNA spanning the open reading frame from constructs was generated by PCR using primers against the T7 promoter (5′-CCCGCGAAATTAATACGACTCACTATAGGG-3′) and terminator (5′-GCTAGTTATTGCTCAGCGG-3′), respectively, followed by PCR clean-up using a Thermo Scientific (Waltham, Massachusetts, U.S.) GeneJET PCR purification kit (catalog number K0701). The linearized DNA was then expressed using the New England BioLabs (Ipswich, Massachusetts, U.S.) PURExpress *in vitro* protein synthesis kit (catalog number E6800L). Briefly, 2.2 μL of linearized DNA was mixed with 0.8 μL [^35^S]-Met, 4 μL solution A and 3 μL solution B, and the mixture was incubated at 37 °C for 15 min shaking at 750 RPM in an Eppendorf Thermomixer. To stop the reaction and precipitate the proteins, 10 μL of 10% trichloroacetic acid was added and the mixture was placed on ice for 30 min. The precipitate was centrifuged at >20,000*g* for 10 min, and then the supernatant was aspirated off. To resuspend the proteins, 12 μL of SDS-sample buffer was added and the reaction tube was shaken for 10 min at 37 °C.

For time-course experiments, reactions were carried out in the same way as above with a few differences. The purified, linearized DNA was adjusted such that the final reaction concentration was 10 ng/μL, as this was found to affect cleavage time. Also, a much larger reaction mixture was made (total 40 μL), from which 5 μL samples were taken at denoted time points and transferred to tubes containing 5 μL of 10% trichloroacetic acid. After the first sample was removed, excess non-radioactive Met was added to a final concentration of 10 mM. Further treatments were the same for all samples.

The PR inhibitor darunavir (Sigma-Aldrich SMLO937) was dissolved at 18 mM in DMSO, and, 0.5 μL was added at the beginning of the PURE reaction where applicable.

For experiments in the presence of purified PR, PURE *in vitro* translation reactions were carried out as above, but with 1 μL of diluted, purified PR added either at the start of the reaction or at the start of the chase, as denoted in the figures. Purified PR (a gift from Dr. Celia Schiffer) was diluted into *in vitro* translation buffer (9 mM Mg (OAc)_2_, 5 mM K-phosphate pH 7.3, 95 mM K-glutamate, 5 mM NH_4_Cl, 0.5 mM CaCl_2_, 1 mM spermidine, 8 mM putrescine, 1 mM DTT) such that the final concentration in each reaction was 1 μM.

Samples (for both single reactions and time-course experiments) were then incubated with 0.25 mg/ml RNase for 30 min. at 37 °C to hydrolyze tRNA, and the proteins were subsequently separated by SDS-PAGE. Gels were fixed in 30% (v/v) methanol or ethanol and 10% (v/v) acetic acid. After incubation in Gel-Dry^™^ Drying Solution (Invitrogen, Thermo Fisher Scientific, Waltham Massachusetts, U.S.) for 30 min, gels were dried in a Hoefer GD 2000 gel dryer (Hoefer Inc., U.S.). After exposing dried gels to phosphorimaging plates (BAS-IP, Fujifilm, Tokyo, Japan) overnight, plates were scanned using a Fuji FLA-9000 imager (Fujifilm, Tokyo, Japan) to collect radio-images of gels. Band intensity profiles were obtained using the Fiji (ImageJ) software [77] and quantified with the in-house software EasyQuant (https://github.com/gvh-lab/easyquant) in order to determine the fraction full-length protein f_FL_ = I_FL_/(I_FL_ + I_A_), where I_A_, I_FL_ are the intensities of the A and FL bands, respectively, Figure 1c. A_c_ and/or FL_c_ size controls were included in the SDS-PAGE to confirm the identity of the *A* and *FL* bands. Each data point in the force profile represents the average of at least three independent replicates (*i.e.*, independent PURE *in vitro* translation reactions) and includes the standard error of the mean (SEM).

Molecular weights were calculated using the Expasy Compute pI/Mw tool at https://www.expasy.org.

To extract lag-time midpoints from pulse-chase experiments, the fraction of cleaved protein *f_cleaved_* = *I_PR_/(I_PR_ + I_A_)* was first determined, where *I_PR_* is the integrated intensity of the released PR, and as above, I_A_ is the integrated intensity of the *A* band, Figure S1. The resulting *f_cleaved_* as a function of chase time was fit to a simple two-state sigmoidal curve:

(1) $$f_{cleaved}=\frac{1}{1+e^{-k\cdot \left(t-t_{0.5}\right)}}$$

where *k* is the slope of the transition and *t*_0.5_ is the lag-time midpoint Supplemental Figure S2.

To determine arrest-peptide release rates (*k*_*R*_) and release-time midpoints $\left(t_{0.5}^{R}\right)$, the fraction arrested $f_{A}(t)\;\left(=1-f_{FL}(t)\right)$ was fit to a single exponential decay function,

(2) $$f_{A}(t)=f_{A}(0)\cdot e^{-k_{R}\cdot t}$$

where *f_A_(t)* is the fraction of arrested protein at chase time *t*, and *f_A_*(0) is the fraction of arrested at time zero (*i.e.*, at the beginning of the chase). Pulse-chase curves were fit using homemade Python scripts invoking SciPy [78] non-linear least squares fitting. Arrest peptide release-time mid-points were calculated as $t_{0.5}^{R}=-\ln(0.5)/k_{R}$.

### Pulse proteolysis

PURE *in vitro* transcription/translation reactions were carried out as above for 15 min in the presence of excess [^35^S]-Met. Reactions were stopped by the addition of chloramphenicol to a final concentration of 3.3 mM. Reactions were then split into equal parts to receive either thermolysin or buffer. Proteolysis was performed by adding thermolysin (suspended in 50 mM Tris and 500 μM ZnCl2, pH 7.0) to a final concentration of 0.75 mg/mL. Proteolysis was allowed for 1 min at 37 °C in a thermomixer shaking at 750 RPM and stopped by chelation with a final concentration of 100 mM EDTA. Next, equal volumes of ice-cold 10% TCA were added, and the precipitation, resuspension, SDS-PAGE, gel drying, and visualization continued as above.

## Supplementary Material

Suppl Figs

Suppl Table

## Data Availability

Python scripts used for fitting pulse-chase curves are available upon reasonable request.

## Appendix A. Supplementary material

Supplementary material to this article can be found online at https://doi.org/10.1016/j.jmb.2026.169788.
