# Supplementary material for: Cotranslational Folding and “Constrained Monomers” in the Maturation of HIV-1 Protease
Source: J Mol Biol. Author manuscript; Available in PMC 2026 Jul 28. (PMC13411501; doi:10.1016/j.jmb.2026.169788)
Supplement: Suppl Figs [file NIHMS2192590-supplement-Suppl_Figs.pdf]

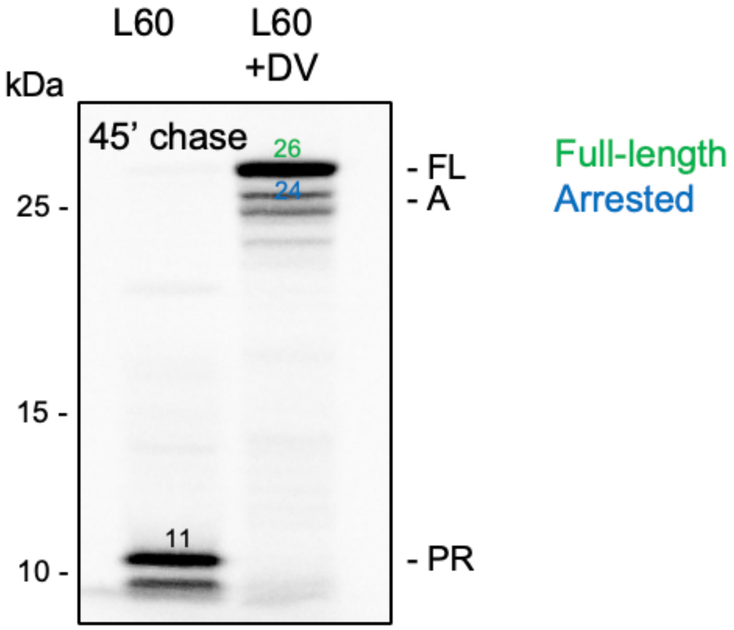

Supplemental Figure S1. Effect of darunavir (DV) on the autoproteolysis of the L60 construct. L60 was translated *in vitro* in the presence of [^{35}S]-Met for 15 min. and then chased in the presence of excess non-radioactive Met for 45 min, in the absence and presence (+DV) of 850 µM darunavir. Equal concentrations of DMSO were included in the ±DV reactions. Mw's are indicated, color-coded as in Fig. 2a.

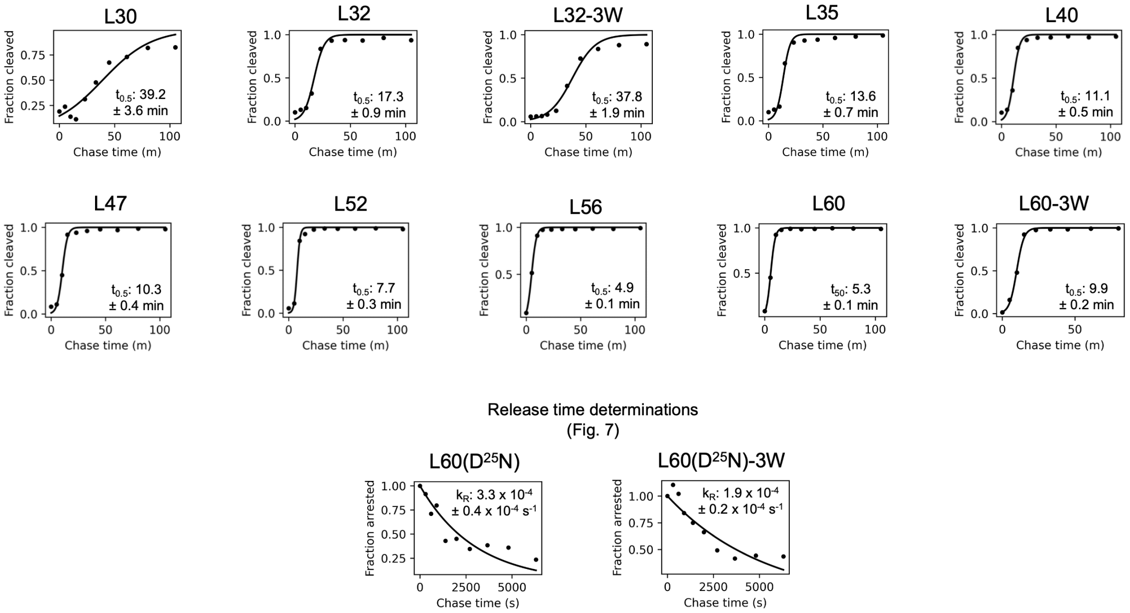

Supplemental Figure S2. *Determination of lag-time* ($t_{0.5}$) *and release-time* ($t_{0.5}^{R}$) *midpoints.* The intensities of bands representing the arrested fraction and the mature PR in Fig. 4 were fit to a two-state sigmoidal equation to determine $t_{0.5}$ values and standard errors. For $D^{25}N$ constructs, plots and fits used to determine $t_{0.5}^{R}$ values are shown ± the standard error of the fitted value. See Methods for details.

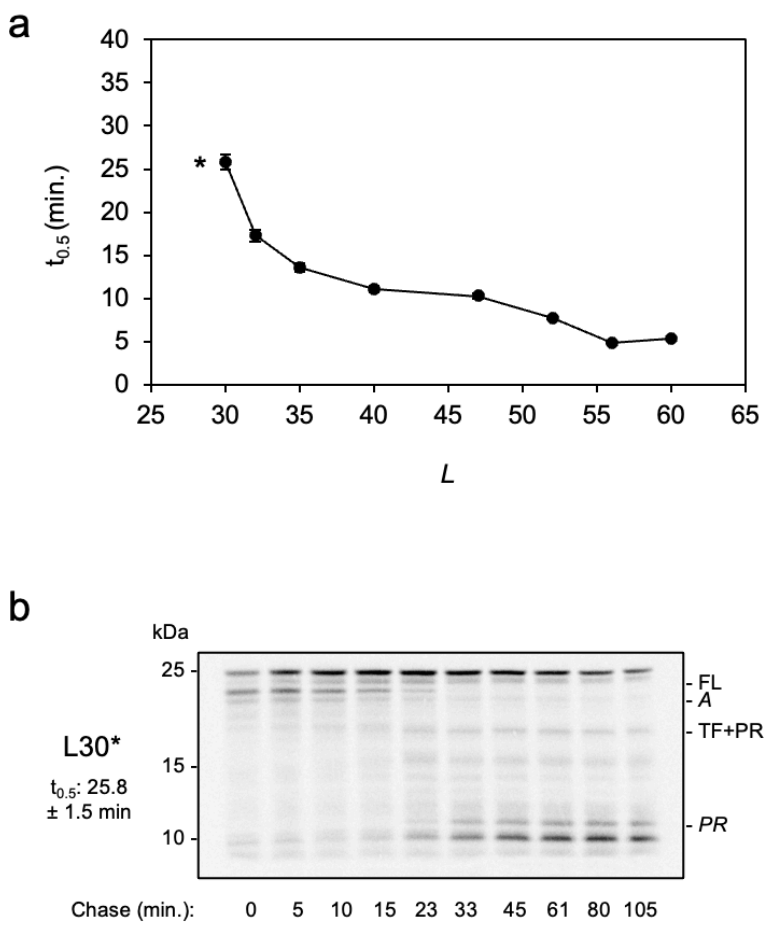

Supplemental Figure S3. *Lag-time midpoints vary with L.* (a) $t_{0.5}$ values for the constructs shown in Fig. 4. The value for *L* = 30 residues (indicated by *) is for the construct shown in panel *b*. (b) Pulse-chase analysis (15 min. pulse) of the mutated L30* construct with an active PR/RT cleavage site (TLNF/PISG).

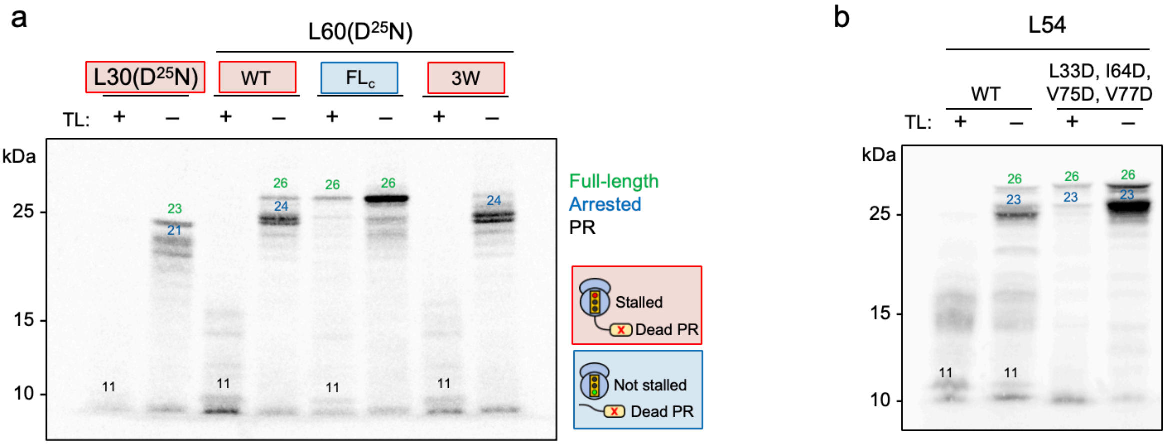

Supplemental Figure S4. *Pulse-proteolysis with thermolysin.* (a) Constructs L30(D^{25}N), L60(D^{25}N), L60(D^{25}N)[FL_c], and L60(D^{25}N)-3W were translated in the presence of [^{35}S]-Met for 15 min. in the PURE system and then incubated with thermolysin (+TL, 0.75 mg/mL final concentration) or buffer (-TL) for 1 min. (b) Same as in panel *a*, but for constructs L54 and L54(L^{33}D+I^{64}D+V^{75}D+V^{77}D).
