# Supplementary material for: Cotranslational Folding and “Constrained Monomers” in the Maturation of HIV-1 Protease
Source: J Mol Biol. Author manuscript; Available in PMC 2026 Jul 28. (PMC13411501; doi:10.1016/j.jmb.2026.169788)
Supplement: Suppl Table [file NIHMS2192590-supplement-Suppl_Table.pdf]

Supplemental Table S1

TF/PR/RT-linker-HA-AP-tail sequence for all constructs. The PR part is underlined, a short linker is in grey, and the SecM AP is in cyan. TF/PR and PR/RT cleavage sites are highlighted in yellow. * signifies a stop codon. Met residues are marked in red.

L60

MFFREDLAFPQGKAREFSSEQTRANSPTRRELQVWGRDNNSLSEAGADRQGTVSFSF/PQITLWQRPLVTIKIGG
QLKEALLDTGADDTVLEEMNLPGRWKPKMIGGIGGFIKVRQYDQILIEICGHKAIGTVLVGPTPVNIIGRNLLTQ
IGCTLNF/PISPIETVPVKLKPGMDGPKVKQWPLTEEKSGSGYPYDVPDYAFSTPVWISQAQGIRAGPGSSDKQE
GEWPTGLRLSRIGGIH*

L60 (D^{25}N)

MFFREDLAFPQGKAREFSSEQTRANSPTRRELQVWGRDNNSLSEAGADRQGTVSFSF/PQITLWQRPLVTIKIGG
QLKEALL*N*TGADDTVLEEMNLPGRWKPKMIGGIGGFIKVRQYDQILIEICGHKAIGTVLVGPTPVNIIGRNLLTQ
IGCTLNF/PISPIETVPVKLKPGMDGPKVKQWPLTEEKSGSGYPYDVPDYAFSTPVWISQAQGIRAGPGSSDKQE
GEWPTGLRLSRIGGIH*

L60-3W

MFFREDLAFPQGKAREFSSEQTRANSPTRRELQVWGRDNNSLSEAGADRQGTVSFSF/PQITLWQRPLVTIKIGG
QLKEALLDTGADDTVLEEMNLPGRWKPKMIGGIGGFIKVRQYDQILIEICGHKAIGTVLVGPTPVNIIGRNLLTQ
IGCTLNF/PISPIETVPVKLKPGMDGPKVKQWPLTEEKSGSGYPYDVPDYAFSTPVWIWWWPPIRGSPGSSDKQE
GEWPTGLRLSRIGGIH*

L60 (D^{25}N) -3W

MFFREDLAFPQGKAREFSSEQTRANSPTRRELQVWGRDNNSLSEAGADRQGTVSFSF/PQITLWQRPLVTIKIGG
QLKEALL*N*TGADDTVLEEMNLPGRWKPKMIGGIGGFIKVRQYDQILIEICGHKAIGTVLVGPTPVNIIGRNLLTQ
IGCTLNF/PISPIETVPVKLKPGMDGPKVKQWPLTEEKSGSGYPYDVPDYAFSTPVWIWWWPPIRGSPGSSDKQE
GEWPTGLRLSRIGGIH*

L58

MFFREDLAFPQGKAREFSSEQTRANSPTRRELQVWGRDNNSLSEAGADRQGTVSFSF/PQITLWQRPLVTIKIGG
QLKEALLDTGADDTVLEEMNLPGRWKPKMIGGIGGFIKVRQYDQILIEICGHKAIGTVLVGPTPVNIIGRNLLTQ
IGCTLNF/PISPIETVPVKLKPGMDGPKVKQWPLTESGSGYPYDVPDYAFSTPVWISQAQGIRAGPGSSDKQEGE
WPTGLRLSRIGGIH*

L56

MFFREDLAFPQGKAREFSSEQTRANSPTRRELQVWGRDNNSLSEAGADRQGTVSFSF/PQITLWQRPLVTIKIGG
QLKEALLDTGADDTVLEEMNLPGRWKPKMIGGIGGFIKVRQYDQILIEICGHKAIGTVLVGPTPVNIIGRNLLTQ
IGCTLNF/PISPIETVPVKLKPGMDGPKVKQWPLSGSGYPYDVPDYAFSTPVWISQAQGIRAGPGSSDKQEGEWP
TGLRLSRIGGIH*

L54

MFFREDLAFPQGKAREFSSEQTRANSPTRRELQVWGRDNNSLSEAGADRQGTVSFSF/PQITLWQRPLVTIKIGG
QLKEALLDTGADDTVLEEMNLPGRWKPKMIGGIGGFIKVRQYDQILIEICGHKAIGTVLVGPTPVNIIGRNLLTQ
IGCTLNF/PISPIETVPVKLKPGMDGPKVKQWSGSGYPYDVPDYAFSTPVWISQAQGIRAGPGSSDKQEGEWPTG
LRLSRIGGIH*

L54 (L^{33}D+I^{64}D+V^{75}D+V^{77}D)

MFFREDLAFPQGKAREFSSEQTRANSPTRRELQVWGRDNNSLSEAGADRQGTVSFSF/PQITLWQRPLVTIKIGG
QLKEALLDTGADDTV*D*EEMNLPGRWKPKMIGGIGGFIKVRQYDQIL*D*EICGHKAIGT*D*L*D*GPTPVNIIGRNLLTQ
IGCTLNF/PISPIETVPVKLKPGMDGPKVKQWSGSGYPYDVPDYAFSTPVWISQAQGIRAGPGSSDKQEGEWPTG
LRLSRIGGIH*

L52

MFFREDLAFPQGKAREFSSEQTRANSPTRRELQVWGRDNNSLSEAGADRQGTVSFSF/PQITLWQRPLVTIKIGG
QLKEALLDTGADDTVLEEMNLPGRWKPKMIGGIGGFIKVRQYDQILIEICGHKAIGTVLVGPTPVNIIGRNLLTQ
IGCTLNF/PISPIETVPVKLKPGMDGPKVKSGSGYPYDVPDYAFSTPVWISQAQGIRAGPGSSDKQEGEWPTGLR
LSRIGGIH*

L50

MFFREDLAFPQGKAREFSSEQTRANSPTRRELQVWGRDNNSLSEAGADRQGTVSFSF/PQITLWQRPLVTIKIGG
QLKEALLDTGADDTVLEEMNLPGRWKPKMIGGIGGFIKVRQYDQILIEICGHKAIGTVLVGPTPVNIIGRNLLTQ
IGCTLNF/PISPIETVPVKLKPGMDGPKSGSGYPYDVPDYAFSTPVWISQAQGIRAGPGSSDKQEGEWPTGLRLS
RIGGIH*

L47

MFFREDLAFPQGKAREFSSEQTRANSPTRRELQVWGRDNNSLSEAGADRQGTVSFSF/PQITLWQRPLVTIKIGG
QLKEALLDTGADDTVLEEMNLPGRWKPKMIGGIGGFIKVRQYDQILIEICGHKAIGTVLVGPTPVNIIGRNLLTQ
IGCTLNF/PISPIETVPVKLKPGMDSGSGYPYDVPDYAFSTPVWISQAQGIRAGPGSSDKQEGEWPTGLRLSRIG
GIH*

L45

MFFREDLAFPQGKAREFSSEQTRANSPTRRELQVWGRDNNSLSEAGADRQGTVSFSF/PQITLWQRPLVTIKIGG
QLKEALLDTGADDTVLEEMNLPGRWKPKMIGGIGGFIKVRQYDQILIEICGHKAIGTVLVGPTPVNIIGRNLLTQ
IGCTLNF/PISPIETVPVKLKPGSGSGYPYDVPDYAFSTPVWISQAQGIRAGPGSSDKQEGEWPTGLRLSRIGGI
H*

L45 (D^{25}N)

MFFREDLAFPQGKAREFSSEQTRANSPTRRELQVWGRDNNSLSEAGADRQGTVSFSF/PQITLWQRPLVTIKIGG
QLKEALL*N*TGADDTVLEEMNLPGRWKPKMIGGIGGFIKVRQYDQILIEICGHKAIGTVLVGPTPVNIIGRNLLTQ
IGCTLNF/PISPIETVPVKLKPGSGSGYPYDVPDYAFSTPVWISQAQGIRAGPGSSDKQEGEWPTGLRLSRIGGI
H*

L42

MFFREDLAFPQGKAREFSSEQTRANSPTRRELQVWGRDNNSLSEAGADRQGTVSFSF/PQITLWQRPLVTIKIGG
QLKEALLDTGADDTVLEEMNLPGRWKPKMIGGIGGFIKVRQYDQILIEICGHKAIGTVLVGPTPVNIIGRNLLTQ
IGCTLNF/PISPIETVPVKLSGSGYPYDVPDYAFSTPVWISQAQGIRAGPGSSDKQEGEWPTGLRLSRIGGIH*

L40

MFFREDLAFPQGKAREFSSEQTRANSPTRRELQVWGRDNNSLSEAGADRQGTVSFSF/PQITLWQRPLVTIKIGG
QLKEALLDTGADDTVLEEMNLPGRWKPKMIGGIGGFIKVRQYDQILIEICGHKAIGTVLVGPTPVNIIGRNLLTQ
IGCTLNF/PISPIETVPVSGSGYPYDVPDYAFSTPVWISQAQGIRAGPGSSDKQEGEWPTGLRLSRIGGIH*

L37

MFFREDLAFPQGKAREFSSEQTRANSPTRRELQVWGRDNNSLSEAGADRQGTVSFSF/PQITLWQRPLVTIKIGG
QLKEALLDTGADDTVLEEMNLPGRWKPKMIGGIGGFIKVRQYDQILIEICGHKAIGTVLVGPTPVNIIGRNLLTQ
IGCTLNF/PISPIETSGSGYPYDVPDYAFSTPVWISQAQGIRAGPGSSDKQEGEWPTGLRLSRIGGIH*

L37 (D^{25}N)

MFFREDLAFPQGKAREFSSEQTRANSPTRRELQVWGRDNNSLSEAGADRQGTVSFSF/PQITLWQRPLVTIKIGG
QLKEALL*N*TGADDTVLEEMNLPGRWKPKMIGGIGGFIKVRQYDQILIEICGHKAIGTVLVGPTPVNIIGRNLLTQ
IGCTLNF/PISPIETSGSGYPYDVPDYAFSTPVWISQAQGIRAGPGSSDKQEGEWPTGLRLSRIGGIH*

L35

MFFREDLAFPQGKAREFSSEQTRANSPTRRELQVWGRDNNSLSEAGADRQGTVSFSF/PQITLWQRPLVTIKIGG
QLKEALLDTGADDTVLEEMNLPGRWKPKMIGGIGGFIKVRQYDQILIEICGHKAIGTVLVGPTPVNIIGRNLLTQ
IGCTLNF/PISPISGSGYPYDVPDYAFSTPVWISQAQGIRAGPGSSDKQEGEWPTGLRLSRIGGIH*

L32

MFFREDLAFPQGKAREFSSEQTRANSPTRRELQVWGRDNNSLSEAGADRQGTVSFSF/PQITLWQRPLVTIKIGG
QLKEALLDTGADDTVLEEMNLPGRWKPKMIGGIGGFIKVRQYDQILIEICGHKAIGTVLVGPTPVNIIGRNLLTQ
IGCTLNF/PISGSGYPYDVPDYAFSTPVWISQAQGIRAGPGSSDKQEGEWPTGLRLSRIGGIH*

L32-3W

MFFREDLAFPQGKAREFSSEQTRANSPTRRELQVWGRDNNSLSEAGADRQGTVSFSF/PQITLWQRPLVTIKIGG
QLKEALLDTGADDTVLEEMNLPGRWKPKMIGGIGGFIKVRQYDQILIEICGHKAIGTVLVGPTPVNIIGRNLLTQ
IGCTLNF/PISGSGYPYDVPDYAFSTPVWIWWWPPIRGSPGSSDKQEGEWPTGLRLSRIGGIH*

L30

MFFREDLAFPQGKAREFSSEQTRANSPTRRELQVWGRDNNSLSEAGADRQGTVSFSF/PQITLWQRPLVTIKIGG
QLKEALLDTGADDTVLEEMNLPGRWKPKMIGGIGGFIKVRQYDQILIEICGHKAIGTVLVGPTPVNIIGRNLLTQ
IGCTLNFSGSGYPYDVPDYAFSTPVWISQAQGIRAGPGSSDKQEGEWPTGLRLSRIGGIH*

L30*

MFFREDLAFPQGKAREFSSEQTRANSPTRRELQVWGRDNNSLSEAGADRQGTVSFSF/PQITLWQRPLVTIKIGG
QLKEALLDTGADDTVLEEMNLPGRWKPKMIGGIGGFIKVRQYDQILIEICGHKAIGTVLVGPTPVNIIGRNLLTQ
IGCTLNF/PISGYPYDVPDYAFSTPVWISQAQGIRAGPGSSDKQEGEWPTGLRLSRIGGIH*

L30 (D^{25}N)

MFFREDLAFPQGKAREFSSEQTRANSPTRRELQVWGRDNNSLSEAGADRQGTVSFSF/PQITLWQRPLVTIKIGG
QLKEALL*N*TGADDTVLEEMNLPGRWKPKMIGGIGGFIKVRQYDQILIEICGHKAIGTVLVGPTPVNIIGRNLLTQ
IGCTLNFSGSGYPYDVPDYAFSTPVWISQAQGIRAGPGSSDKQEGEWPTGLRLSRIGGIH*

L28

MFFREDLAFPQGKAREFSSEQTRANSPTRRELQVWGRDNNSLSEAGADRQGTVSFSF/PQITLWQRPLVTIKIGG
QLKEALLDTGADDTVLEEMNLPGRWKPKMIGGIGGFIKVRQYDQILIEICGHKAIGTVLVGPTPVNIIGRNLLTQ
IGCTLNFSGYPYDVPDYAFSTPVWISQAQGIRAGPGSSDKQEGEWPTGLRLSRIGGIH*

L26

MFFREDLAFPQGKAREFSSEQTRANSPTRRELQVWGRDNNSLSEAGADRQGTVSFSF/PQITLWQRPLVTIKIGG
QLKEALLDTGADDTVLEEMNLPGRWKPKMIGGIGGFIKVRQYDQILIEICGHKAIGTVLVGPTPVNIIGRNLLTQ
IGCTLNFYPYDVPDYAFSTPVWISQAQGIRAGPGSSDKQEGEWPTGLRLSRIGGIH*

L24

MFFREDLAFPQGKAREFSSEQTRANSPTRRELQVWGRDNNSLSEAGADRQGTVSFSF/PQITLWQRPLVTIKIGG
QLKEALLDTGADDTVLEEMNLPGRWKPKMIGGIGGFIKVRQYDQILIEICGHKAIGTVLVGPTPVNIIGRNLLTQ
IGCTLYPYDVPDYAFSTPVWISQAQGIRAGPGSSDKQEGEWPTGLRLSRIGGIH*

L22

MFFREDLAFPQGKAREFSSEQTRANSPTRRELQVWGRDNNSLSEAGADRQGTVSFSF/PQITLWQRPLVTIKIGG
QLKEALLDTGADDTVLEEMNLPGRWKPKMIGGIGGFIKVRQYDQILIEICGHKAIGTVLVGPTPVNIIGRNLLTQ
IGCYPYDVPDYAFSTPVWISQAQGIRAGPGSSDKQEGEWPTGLRLSRIGGIH*

L20

MFFREDLAFPQGKAREFSSEQTRANSPTRRELQVWGRDNNSLSEAGADRQGTVSFSF/PQITLWQRPLVTIKIGG
QLKEALLDTGADDTVLEEMNLPGRWKPKMIGGIGGFIKVRQYDQILIEICGHKAIGTVLVGPTPVNIIGRNLLTQ
IYPYDVPDYAFSTPVWISQAQGIRAGPGSSDKQEGEWPTGLRLSRIGGIH*

L18

MFFREDLAFPQGKAREFSSEQTRANSPTRRELQVWGRDNNSLSEAGADRQGTVSFSF/PQITLWQRPLVTIKIGG
QLKEALLDTGADDTVLEEMNLPGRWKPKMIGGIGGFIKVRQYDQILIEICGHKAIGTVLVGPTPVNIIGRNLLTY
PYDVPDYAFSTPVWISQAQGIRAGPGSSDKQEGEWPTGLRLSRIGGIH*
